# Thyroid Stimulating Hormone Receptor

**DOI:** 10.4274/2017.26.suppl.10

**Published:** 2017-01-09

**Authors:** Murat Tuncel

**Affiliations:** 1 Hacettepe University Faculty of Medicine, Department of Nuclear Medicine, Ankara, Turkey

**Keywords:** Thyroid stimulating hormone receptor, genetic, epigenetic alteration, thyroid cancer, adenoma

## Abstract

Thyroid stimulating hormone receptor (TSHR) plays a pivotal role in thyroid hormone metabolism. It is a major controller of thyroid cell function and growth. Mutations in TSHR may lead to several thyroid diseases, most commonly hyperthyroidism. Although its genetic and epigenetic alterations do not directly lead to carcinogenesis, it has a crucial role in tumor growth, which is initiated by several oncogenes. This article will provide a brief review of TSHR and related diseases.

## Abstract (tr)

Tiroid stimulan hormon reseptörü (TSHR) tiroid hormon metabolizmasında merkezi bir rol oynar. Tiroid hücrelerinin fonksiyonlarının ve büyümesinin kontrolünde yardımcıdır. TSHR mutasyonları bir dizi tiroid hastalığına, en çok hipertiroidizme neden olmaktadır. Her ne kadar bu reseptörün genetik ve epigenetik değişiklikleri direkt olarak karsinogeneze yol açmasa da, farklı onkogenler tarafından başlatılmış tümörün büyümesinde ciddi bir rol oynar. Bu makalede TSHR ve ilişkili hastalıklar özetlenmektedir.

## INTRODUCTION

Thyroid stimulating hormone receptor (TSHR) has been first cloned in 1989. It is located on chromosome 14q and contains 10 exons (1). It encodes the synthesis of a protein with 764 amino acids, and has a molecular weight of 87 kDa. The first 9 exons of the gene encode a large amino-terminal ectodomain, while exon 10 encodes seven transmembrane segments and a intracytoplasmic domain with a carboxyl-terminal segment. The long amino-terminal segment of the receptor creates high affinity for TSH binding. TSHR is divided into two subunits (α and β) by post-translational proteolysis and each subunit is linked to each other via disulfide bonds. The receptor than undergoes post-translational glycosylation and palmitoylation for full functionality (2).

TSHR belongs to a group of G-protein-coupled seven-transmembrane receptors and is located at the basolateral membrane of thyroid follicular cells (2). Studies suggest that several G protein subtypes are involved in the signal transfer, but Gαs and Gαq have been shown to be the major subtypes that mediate TSHR signals (3). TSHR activation results in intracellular signaling via G proteins that modulate the effector molecule activity. Among these; Gs protein leads to activation of the cyclic adenosine monophosphate (AMP) cascade, and the Gq protein activates the phospholipase C (PLC) cascade. At higher TSH concentrations, cAMP binds to protein kinase A (PKA), which phosphorylates different effectors with its enhanced catalytic activity. Inositol 1,4,5-triphosphate and diacylglycerol are generated by activated PLC. These molecules stimulate the release of Ca^2+^ into the cytoplasm and activate the protein kinase C (PKC) pathway. Increased levels of intracellular Ca^2+^ and PLC activity play a major role in the regulation of H_2_O_2_ production, thyroglobulin (Tg) iodination and iodide efflux, while adenylate cyclase and cAMP regulate transcription of sodium-iodide symporter (NIS), Tg and thyroid peroxidase (TPO), as well as iodide uptake (4,5).

TSH levels positively modulate TSHR in normal cells up to a certain limit, while down regulating TSHR at high concentrations (6). Over-activation of the cAMP pathway by chronic TSHR stimulation causes excess hormone secretion and thyroid hyperplasia, which results in clinical hyperthyroidism. Increased secretion of the thyroid hormone than leads to negative feedback at the hypothalamic-pituitary level resulting in suppressed TSH secretion. This mechanism may be clearly detected by scintigraphic methods in a hyperthyroid patient with suppressed TSH levels. Images show decreased radiotracer uptake in normal parts of the thyroid gland due hot toxic adenoma with high radiotracer uptake. The uptake by the normal thyroid gland returns to normal levels following ablation of toxic adenoma, while the TSH levels also return to normal (7).

## THYROID STIMULATING HORMONE RECEPTOR MUTATIONS IN BENIGN DISEASES

Several mutations may occur in the TSHR gene that influence either the protein component or post-translational modifications of the receptor. The mutations may be activating (constitutive) or deactivating. These are dominant mutations and modification in one allele is sufficient for generating the abnormal phenotype. TSHR mutations are defined in several diseases like familial gestational hyperthyroidism, autonomous toxic adenomas, hereditary or sporadic toxic thyroid hyperplasia, familial non-autoimmune hyperthyroidism, Graves’ disease and autoimmune hypothyroidism (8,9). Autosomal dominant non-autoimmune hyperthyroidism may be caused by germ-line TSHR mutations, and de novo mutations may lead to sporadic non-autoimmune hyperthyroidism.

Somatic activating mutations of the TSHR or Gsα proteins constitutively activate the cAMP pathway. This activation causes clonal autonomous growth and hyper-functioning of the thyroid follicular cells which results in a toxic adenoma. Cells with activating mutation may have an increased expression of the NIS, which is seen as a high uptake or ‘hot nodule’ image on scintigraphy (10). The prevalence of TSH receptor mutations in toxic adenomas varies in different studies, but is reported to be as high as 80%. Differences in iodine intake, sampling technique, and methodological approaches might explain this variance (11). Activating mutations are mainly located in the β subunit of the TSHR. However, Kopp et al. (12) reported constitutive activation to the receptor, caused by substitutions at serine 281 (S281I/N/T), which is a residue located in the extracellular α subunit. The exact mechanisms that result in function gain is not clearly understood. It has been suggested that mutations may alter the configuration of the transmembrane segments, mimicking the structural changes occurred after binding of ligand or alternatively some mutations may change the structure of the domains that inhibit receptor coupling to G proteins in the absence of TSH (8,13).

In contrast to hyper-functioning nodules, cold hypo-functioning nodules have a low incidence of mutations. Mutations in Gs protein were detected in 27% of nonfunctioning adenomas in one series, however, this was not verified by others. These nodules are believed to have mutations of genes linked with de-differentiation. Activating mutations in the RAS proto-oncogene pathway have been detected in 20% of thyroid adenomas with frequencies similar to those found in follicular thyroid (FTC) and papillary thyroid carcinomas (PTC) (11).

## THYROID STIMULATING HORMONE RECEPTOR AND GS GENE MUTATIONS IN THYROID CARCINOMAS

Mutations of Gsa subunit and the TSHR gene rarely occur in well-differentiated thyroid cancers. Although activated cAMP pathway results in enhanced growth, it is not sufficient for malignant transformation of normal thyrocytes. Based on available data, TSHR and Gs gene mutations are not involved in carcinogenesis, except in a small proportion (<6%) of cases (14,15). However, in thyroid carcinomas with a poor response to TSH and high basal adenylate cyclase activity, mutations in TSHR and Gs were reported in 12% of FTC and in 13% of PTC (16).

TSHR mutations were also reported in malignant hot nodules at scintigraphy. Niepomniszcze et al. (17) reported a case of FTC presenting as a hot nodule. Sequence analysis revealed a constitutive mutation at codon 620 of the TSHR gene and a G12C Ki-RAS mutation. It has been reported that RAS mutation could be the driver for transformation, since hot nodules only rarely progress to carcinoma. Gozu et al. (18) described a TSHR mutation in a PTC presenting as a hot nodule, and a similar finding was observed by Camacho et al. (19) in a FTC. Finally, Russo and colleagues described an autonomously functioning Hurthle cell carcinoma with a TSHR mutation and absence of either RAS or TP53 mutations (20). According to these observations, screening of mutations in different oncogenes related to thyroid cancer and the role of TSHR mutations in transformation was not well established. From the limited data available, it seems that activation of the cAMP pathway does not a major role in cell transformation. Most hyper-functioning tumors harbor both TSHR mutations and proto-oncogene mutations; this coexistence suggests that carcinomas arise from the activity of classical oncogenes, such as RAS and RET/PTC, and that the TSHR and Gs mutations contribute to the hyper-functioning features of the neoplasms.

## THYROID STIMULATING HORMONE RECEPTOR PATHWAY, RELATIONS WITH OTHER GENETIC AND EPIGENETIC ALTERATIONS

Several pathways are responsible for tumor carcinogenesis in thyroid cancer. Multiple genetic and epigenetic alterations that lead to activation of the mitogen-activated protein kinases and phosphatidylinositol-3-kinase-AKT signaling pathways are required for the development and progression of thyroid cancer. Common genetic alterations found in thyroid cancer include point mutation of the BRAF and RAS genes (seen up to 45% of patients) as well as RET/PTC and PAX8/PPARγ chromosomal rearrangements (21). Ionizing radiation, chemical mutagenesis and dietary iodine excess were suggested as etiologic factors of these chromosomal rearrangements and mutations (22). As described earlier, TSH-TSHR signaling pathway plays a critical role for thyroid cell growth and proliferation. It acts via common pathways as other oncogenes and has a role in controlling cell growth and carcinogenesis. Several animal studies were performed to evaluate the role of TSHR signaling pathway and its relation with other oncogenes in thyroid cancer. Lu et al. (23) used a special mouse model (TRβ^PV^/^PV^ mice) that has a negative mutation (PV) of the thyroid hormone-β receptor (TRβ). These mice have elevated TSH and serum thyroid hormone levels, and they spontaneously develop FTC. The authors observed that when these mice were crossed with TSH receptor gene knockout TSHR−/− mice, and these mice did not develop thyroid cancer. This study demonstrated the requirement of TSH-TSHR signaling pathway in thyroid carcinogenesis in this mouse model. Similarly, in thyroid-specific knock-in of BRAFV600E LSL-Braf(V600E)/TPO-Cre) mouse model in which mice develop aggressive PTC, crossing of these mice with TSHR−/− mice blocked the development of thyroid cancer (24). However, it is not clearly demonstrated if THSH-TSHR signaling is essential for the initiation of thyroid cancer or if it is required for the TSHR-dependent generation and growth of oncogene-stimulated thyroid cancer cells. Over-activation of TSH-TSHR pathway through activating mutations in TSHR or Gs_α_ is known to cause benign hyper-functional FT; however, these tumors almost never undergo malignant transformation. This suggests that TSHR signaling may be protective against malignant transformation of thyroid cells. TSHR may avoid malignant transformation of thyroid cells and suppress the occurrence of thyroid cancer, but it may promote the growth and progression of thyroid cancer once it has been initiated by oncogenic modifications. TSHR expression is also related with other thyroid specific genes. Presence of TSHR gene expression effects other thyroid specific genes. In their study, Feng et al. (25) showed that after transfection of recombinant plasmid pcDNA3.1-hTSHR into dedifferentiated FTC-133 cells, the ^125^I uptake, TSHR, NIS, TPO and Tg mRNAs were significantly increased by 2, 9, 1.7, 4, 1.5 and 2.2 times, respectively, as compared to control levels. The authors concluded that decreased TSHR expression correlated with FTC-133 dedifferentiation, and TSHR transfection contributed to the re-differentiation of these FTC cells. Based on these studies, it can be suggested that TSHR is needed in early progression of the disease and that it is not required after de-differantiation (contrary it re-induce cell differentiation).

The most common and well recognized genetic alteration in thyroid cancer is BRAF(V600E) mutation, which is present in up to 45% of thyroid malignancies and in up to 62% of radioactive iodine-resistant thyroid tumors. This mutation is associated with down regulation of several thyroid specific genes. Kleiman et al. (26) evaluated the effect of BRAF inhibition and TSH supplementation on (131)I uptake in BRAF(V600E)-mutant (WRO) human thyroid cancer cells. Transfection of WRO cells with small interfering RNA targeting BRAF causes an increase in expression of the NIS gene by 5.5-fold and the TSHR gene by 2.8-fold (p=0.02). This increase was also noted in NIS and TSHR protein levels. The effect of BRAF inhibition was also TSH dependent and not detected in case of TSH depletion. In their study Durante et al. (27) characterized the expression of thyroid-specific genes associated with BRAF mutation. mRNA levels for NIS, apical iodide transporter (AIT-B), Tg, TPO, TSHR, the transcription factor PAX8, and glucose transporter type 1 (Glut1) were measured, and these levels for all thyroid-specific genes were reported to be reduced in all PTCs vs. normal thyroid tissues. NIS, AIT-B, Tg, and TPO expression was significantly lower in BRAF-mut tumors than in the BRAF-wt group. However, in this study, TSHR expression was not significantly effected by BRAF mutation status. Glut-1 transcript levels were increased in all PTCs, and additional increases were noted in BRAF-mut tumors. Authors stated that BRAF^V600E^ mutation in PTCs was associated with reduced expression of key genes involved in iodine metabolism and that this may suggest a more aggressive tumor as can be predicted by an increase in Glut-1 transcript levels.

In addition to mutations, the age of the patient was also important for thyroid specific gene expression in thyroid tumors. Espadinha et al. (28) have found that among PTCs, the mean expression of Pendred syndrome gene (PDS), TPO and TSH-R was significantly lower in the elderly. The finding of higher PDS, TPO and TSH-R mRNA expression in pediatric vs. adult primary tumor tissues supports the hypothesis that this might contribute to the increased functional activity of metastases in the pediatric group.

Several epigenetic alterations like DNA methylation and histone modification may also occur in thyroid specific genes. Among these changes, methylation of TSHR is a common form of epigenetic alteration in thyroid cancers and correlates with the presence of other oncogenes. Khan et al. (29) determined methylation of the promoter region of TSHR gene in 25% (15 of 60) of thyroid cancer patients. These patients also had higher TSH levels than the non-methylated patients, suggesting a loss in function of TSHR after methylation. In this study group, BRAF^V600E^ mutation was found in 25 % (15 of 60) patients and within this sub-group the TSHR promoter was methylated in 73.3 % (11 of 15). This study showed the importance of TSHR gene methylation and its significant association with BRAFV600E mutation in thyroid tumors, depicting a positive correlation between TSHR pathway and MAP Kinase pathway. The methylation of TSHR was also confirmed by our group; we showed that after application of DNA methylation inhibitor 5-Azacytidine, TSH-R mRNA expression was increased in both normal thyroid and BCPAP papillary thyroid cancer cell lines. Unfortunately, 5-Azacytidine did not increase radioiodine uptake in the cancer cell line, which suggests that multiple genetic and post-translational alterations are involved in the expression of thyroid specific genes into protein and functional levels (30).

## CONCLUSION

TSHR and its genetic & epigenetic alterations is a stimulating research area that needs further evaluation. It has important correlations with thyroid specific genes, and with several oncogenic pathways in thyroid cancer. Future studies focusing on the modification of genetic and epigenetic alterations of TSHR and the related genes will help better understand the disease process and may lead to a potential cure.
